# Deficiency in the zinc transporter ZIP8 impairs epithelia renewal and enhances lung fibrosis

**DOI:** 10.1172/JCI160595

**Published:** 2022-06-01

**Authors:** Paul S. Foster, Hock L. Tay, Brian G. Oliver

**Affiliations:** 1 School of Biomedical Sciences and Pharmacy, College of Health, Medicine and Wellbeing, University of Newcastle, and Immune Health program, Hunter Medical Research Institute (HMRI), Newcastle, New South Wales, Australia.; 2 School of Life Sciences, University of Technology Sydney, and the Woolcock Institute of Medical Research, University of Sydney, Sydney, New South Wales, Australia.

## Abstract

Although aging and lung injury are linked to the development of idiopathic pulmonary fibrosis (IPF), the underlying pathognomonic processes predisposing to fibrotic lesions remain largely unknown. A deficiency in the ability of type 2 alveolar epithelial cell (AEC2) progenitors to regenerate and repair the epithelia has been proposed as a critical factor. In this issue of the *JCI*, Liang et al. identify a deficiency in the zinc transporter *SLC39A8* (ZIP8) in AEC2s and in the subsequent activation of the sirtuin SIRT1 that predisposes to decreased AEC2 renewal capacity and enhanced lung fibrosis in both IPF and aging lungs. Interestingly, the authors demonstrate the efficacy of modulating dietary zinc levels, suggesting the need for clinical trials to evaluate the therapeutic potential of dietary supplementation and the development of pharmacological modulation of the Zn/ZIP8/SIRT1 axis for treatment.

## Idiopathic pulmonary fibrosis

Most lung diseases are pathologically characterized by fibrosis, which is typically restricted to the airway walls. However, in idiopathic pulmonary fibrosis (IPF), the normal honeycomb structure of alveoli is replaced by fibrotic tissue, resulting in reduced gas exchange in early stages of the disease, which ultimately leads to death from pulmonary failure. IPF is essentially an intractable disease, although some headway has been made with antifibrotics: 5-year cumulative survival rates are 51.4% for people taking antifibrotics versus 43.9% for people not taking these drugs (1), and a 2.5%–4.3% reduction in the decline of forced viral capacity was reported across various antifibrotics in a recent meta-analysis (2). Even with current therapeutics, the prognosis for IPF patients is worse than for most cancers, including lung cancer.

While fibrosis in IPF is of unknown etiology, there are known risk factors for the development of IPF, such as cigarette smoking and lung infections. In the context of lung disease, the cause of fibrosis is contested within the field, with experts divided around the role of inflammation. This debate stems from evidence in diseases such as asthma, where there are data indicating that histopathological changes in the airway wall (termed “airway remodeling”) occurs before the initiation of inflammation; and in IPF, where corticosteroids are ineffective (3). In the context of IPF, another known risk factor is age (4). However, this association is understandable when one considers that the disease occurs in response to environmental exposures. Accelerated cellular aging occurs in IPF and scientifically presents a stronger case for the importance of aging in disease pathogenesis than the actual age of the person (5).

**Figure 1:**
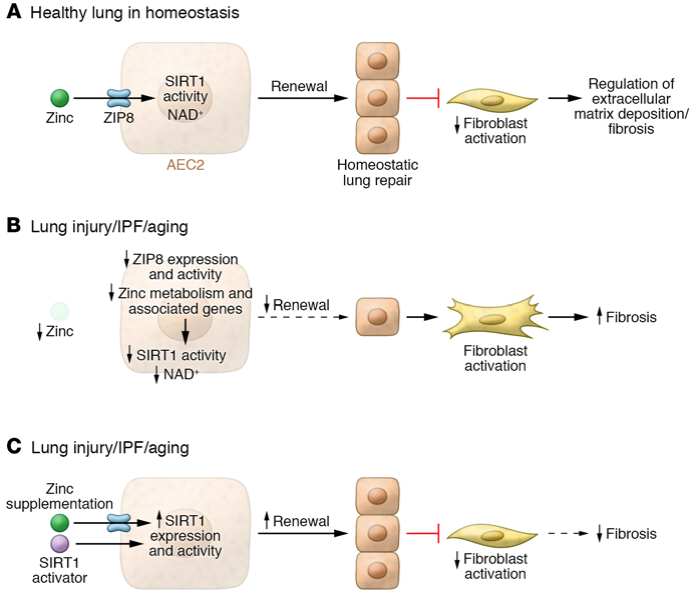
The Zn/ZIP8/SIRT1 axis mediates homeostatic AEC2 progenitor cell renewal and epithelial repair in the lungs. (**A**) In healthy lungs, the Zn/ZIP8/SIRT1 axis regulates homeostatic zinc, NAD^+^ levels, and SIRT1 activation and promotes AEC2 progenitor cell renewal and epithelial repair and turnover, and is a negative regulator of excessive extracellular matrix deposition. (**B**) In IPF and aging lungs, AEC2 zinc and NAD^+^ levels, ZIP8 transporter activity, and SIRT1 expression are reduced. Reduction of these factors results in impaired AEC2 renewal, decreased epithelial repair, and development of fibrotic lesions. (**C**) In IPF and aged lungs, dietary zinc supplementation restores the activity of the Zn/ZIP8/SIRT1 axis and NAD^+^ levels, promoting AEC2 progenitor renewal and inhibiting the development of fibrosis. Pharmacological activation of SIRT1 also restores AEC2 renewal and limits fibrosis.

Alveoli are relatively simple in structure, consisting of type 1 alveolar epithelial cells (AEC1s), endothelial cells, and fibroblasts. AEC1s are terminally differentiated and incapable of division, and as such are continually replaced by AEC2 progenitor cells. A critical factor in the induction of fibrosis is failure to adequately repair epithelial cells after injury (e.g., after smoking or repetitive infections), which leads to overactivation of fibroblasts, excessive extracellular matrix production, and development of fibrotic lesions (6). A deficiency in the regeneration of AEC2s, which act as progenitor cells for the repair process, has been proposed to predispose to the progressive lung fibrosis characteristic of IPF (7, 8). Although impaired AEC2 regeneration is implicated in driving fibrosis, the mechanism underpinning onset remains unknown. Furthermore, elucidation of the disease-inducing pathways may substantially aid in the development of treatments for IPF and other fibrotic lung disorders. In this issue of the *JCI*, Liang et al. (9) identify a deficiency in the zinc transporter *SLC39A8* (ZIP8) in AEC2s as a critical factor predisposing to impaired renewal capacity and enhanced lung fibrosis in both IPF and aging lungs (Figure 1).

## Zinc metabolism and ZIP8 transporter activity

In initial studies, Liang et al. (9) employed unbiased single-cell RNA-Seq (scRNA-Seq) to interrogate the transcriptional activity of IPF AEC2s. These cells were characterized by a failure to regenerate and were characterized as CD31^–^CD45^–^EpCAM^+^ by flow cytometry within the epithelial cell pool. Notably, the cells displayed a decrease in ZIP8 expression, and multiple zinc metabolism–related genes were also downregulated, indicating potential abnormalities in zinc homeostasis. Expression of other AEC2 markers, including SFTPA2, SFTPB, and ABCA3, was also decreased. Maintenance of zinc levels by zinc transporters is critical for homeostatic processes in the lung. Further, zinc deficiency has been linked to decreased progenitor function in the intestinal epithelium, and zinc is known to play a central role in activating the sirtuin signaling pathway, which regulates stem cell maintenance and differentiation (10). Putting pieces of the puzzle together, Liang et al. (9) first demonstrated that IPF AEC2s had lower levels of intracellular zinc than AEC2s from healthy lungs, establishing that zinc metabolism was impaired in IPF. Next, by employing 3D organoid cultures, the authors demonstrated that IPF-derived AEC2 ZIP8^+^ progenitor cells had reduced renewal capacity compared with those from healthy donor cells. Further, both AEC2 populations showed increased renewal capacity in the presence of zinc, and the response was specific to this element.

## The Zn/ZIP8/SIRT1 axis promotes renewal

Next, pathway analysis of the scRNA-Seq data suggested that Sirt1, a critical member of the sirtuin family, may be under the regulation of ZIP8 and contribute to AEC2 progenitor renewal. Importantly, Sirt1 function was downregulated (as determined by activation score and expression) in IPF AEC2s. SIRT1 expression was greater in ZIP8^+^ compared with ZIP8^–^ AEC2s that were isolated from the healthy lung. Treatment of AEC2s with zinc increased the number of SIRT1^+^ cells to a greater extent in healthy than IPF donor lungs, and SIRT was mainly expressed in ZIP8^+^ cells. These data suggested that zinc-induced SIRT1 expression in AEC2s is ZIP8 dependent and downregulation of SIRT1 in IPF AEC2s maybe due to ZIP8 deficiency. Pharmacological activation of SIRT1 demonstrated a direct role for this molecule in progenitor renewal and improved renewal capacity; zinc also amplified this effect, and CRISPR/Cas9 knockout of SIRT1 in A549 cells confirmed the result. Collectively, these studies elegantly demonstrated a critical role for a Zn/ZIP8/SIRT1 axis in promoting renewal capacity in IPF AEC2s (ref. 9 and Figure 1).

## Bleomycin-injured and aging lungs

To further implicate the ZIP8/SIRT1 axis in pathogenesis, Liang et al. (9) employed a bleomycin model of fibrosis in conjunction with aging mice. Isolated AEC2s from bleomycin-injured aged mouse lungs displayed transcriptional changes and phenotypes similar to those of IPF AEC2s. The bleomycin studies also demonstrated that severe loss of ZIP8 function in AEC2s decreased recovery of AEC2 integrity in aged compared with younger lungs after injury. Importantly, AEC2s from aged lungs without injury had reduced ZIP8 expression and decreased renewal capacity relative to AEC2s from lungs of young mice. Zinc treatment also promoted renewal and differentiation of mouse AEC2s; however, this effect was blunted with aging. Thus, loss of ZIP8^+^ AEC2 progenitors might be one of the characteristics of lung aging, and the decreased renewal capacity of aged AEC2s might be due to loss of ZIP8 expression, predisposing to fibrotic lesions.

## Targeting ZIP8 for deletion in AEC2s

In the next series of experiments the final pieces of the puzzle were placed: Liang et al. (9) generated transgenic mice with *Slc39a8* deleted in the adult AEC2 compartment (designated, Zip8^AEC2^) and compared the lungs of 16-week-old Zip8^AEC2^ mice with those of littermate controls. AEC2s of Zip8^AEC2^ mice showed reduced ability to sequester zinc, impaired proliferation in culture, and decreased SIRT1 expression. However, immediate lung inflammation and fibrosis did not develop in Zip8^AEC2^ mice, suggesting that aging or injury required decreased zinc uptake for fibrotic pathology. Indeed, bleomycin-induced injury in 10- to 12-week-old Zip8^AEC2^ mice had fewer AEC2s compared with controls. The transgenic AEC2s had phenotypes similar to those of aged AEC2s taken from 18- to 20-month-old WT mice. scRNA-Seq analysis of AEC2s from young Zip8^AEC2^ mice also demonstrated that aging-related genes were upregulated, identifying zinc/ZIP8 as a potential checkpoint regulator for aging of the lung. Importantly, long-term ZIP8 deletion resulted in spontaneous lung fibrosis in subpleural and interstitial compartments. Bleomycin-induced injury in older Zip8^AEC2^ mice was also more severe. The finding that in older WT mice, a zinc-deficient diet increased bleomycin-induced injury susceptibility and fibrosis and decreased survival rates, while a zinc-supplemented diet decreased lung fibrosis, further highlights the role of zinc (Figure 1).

## Concluding remarks

Liang et al. (9) performed the initial genomic studies on samples taken from only 6 patients and 6 donors, representing 14,687 cells from patients with IPF and 11,381 cells from healthy individuals. However, the authors also leveraged data from other published sources, confirming that *SLC39A8* expression was decreased in AEC2s from individuals with IPF compared with healthy cells, via GEO data sets GSE135893 (11), GSE132915 (12), GSE132771 (13), and GSE128033 (14). These findings support the importance of the functional studies. Furthermore, the decreased Zip8 (*SLC39A8*) expression was not an artifact induced by pretransplantation drug treatments (pirfenidone and nintedanib) administered to patients under investigation. Interestingly, *SLC39A8* was also downregulated in AEC1s from IPF lungs, which warrants further investigation.

Of note, *SLC39A8* levels are higher in human lungs by comparison to other *SLC39* family genes, and lung tissue expresses *SLC39A8* at the highest levels compared with other organs and tissues. Furthermore, *SLC39A8* expression was primarily in EpCAM^+^ positive epithelial cells, indicating that the cells were predominantly AEC2s, and very low in EpCAM^–^ cells, which define mesenchymal cells. Thus, the pulmonary and cellular specificity and expression of this transporter suggest that therapeutic targeting may provide a viable treatment approach for IPF.

Confirmation of the pathogenic role of the Zn/ZIP8/SIRT1 axis in IPF will further support this exciting discovery and focus attention on therapeutic approaches. Further examination of the contribution of other downregulated factors in IPF AEC2s that regulate zinc metabolism (MT1E, MT2A, GCLM, and GSR) may also enhance our understanding of zinc handling in this and other fibrotic diseases. Interestingly, ZIP8-deficient (ZIP8^–^) AEC2s derived from 3D-cultured organoids showed decreased *PDPN* expression, suggesting impaired differentiation. Sirtuin signaling requires both zinc and NAD^+^, and studies have shown that NAD^+^ precursors improve stem cell function and prolong life span (15, 16). Notably, in Liang et al., several genes encoding NAD^+^ synthesis enzymes (e.g., NNMT, NAMPT, KYNU, and NQO1) were downregulated in IPF AEC2s, and the impact of this exciting observation on regulating the Zn/ZIP8/SIRT1 axis and on solving the puzzle of fibrosis remains to be elucidated.

In conclusion, the Liang et al. (9) investigation provides the first evidence to our knowledge of zinc metabolic dysregulation in IPF AEC2s and identifies the zinc transporter *SLC39A8*/ZIP8 as a critical pathogenic factor leading to fibrosis in IPF. It is tantalizing to think that modulation of this and other zinc-regulatory pathways may lead to additional treatments for the debilitating condition of pulmonary fibrosis.

## Version 1 06/01/2022

Electronic publication
